# Strategies for Improving Participation in Diabetes Education. A Qualitative Study

**DOI:** 10.1371/journal.pone.0095035

**Published:** 2014-04-14

**Authors:** Ingmar Schäfer, Marc Pawels, Claudia Küver, Nadine Janis Pohontsch, Martin Scherer, Hendrik van den Bussche, Hanna Kaduszkiewicz

**Affiliations:** 1 Department of Primary Medical Care, University Medical Center Hamburg-Eppendorf, Hamburg, Germany; 2 Institute of General Practice, Medical Faculty, Kiel University, Kiel, Germany; Universitat Rovira i Virgili, Spain

## Abstract

**Objective:**

Diabetes mellitus is highly prevalent and can lead to serious complications and mortality. Patient education can help to avoid negative outcomes, but up to half of the patients do not participate. The aim of this study was to analyze patients' attitudes towards diabetes education in order to identify barriers to participation and develop strategies for better patient education.

**Methods:**

We conducted a qualitative study. Seven GP practices were purposively selected based on socio-demographic data of city districts in Hamburg, Germany. Study participants were selected by their GPs in order to increase participation. Semi-structured face-to-face interviews were conducted with 14 patients. Interviews were audiotaped and transcribed verbatim. The sample size was determined by data saturation. Data were analysed by qualitative content analysis. Categories were determined deductively and inductively.

**Results:**

The interviews yielded four types of barriers: 1) Statements and behaviour of the attending physician influence the patients' decisions about diabetes education. 2) Both, a good state of health related to diabetes and physical/psychosocial comorbidity can be reasons for non-participation. 3) Manifold motivational factors were discussed. They ranged from giving low priority to diabetes to avoidance of implications of diabetes education as being confronted with illness narratives of others. 4) Barriers also include aspects of the patients' knowledge and activity.

**Conclusions:**

First, physicians should encourage patients to participate in diabetes education and argue that they can profit even if actual treatment and examination results are promising. Second, patients with other priorities, psychic comorbidity or functional limitations might profit more from continuous individualized education adapted to their specific situation instead of group education. Third, it might be justified that patients do not participate in diabetes education if they have slightly increased blood sugar values only and no risk for harmful consequences or if they already have sufficient knowledge on diabetes.

## Introduction

Diabetes mellitus is a highly prevalent chronic condition. In Germany, it affected about 8 million people in 2009 which corresponded to 9.8% of the population [1]. Diabetes can lead to serious complications such as cardiovascular disease, nephropathy, retinopathy and lower-extremity amputations. Also, rates of mortality are higher among patients with diabetes compared to the general population [2]. The German health care costs of diabetes are estimated to exceed 48 billion euros in 2009 [1]. The methods of diabetes self-management education in Germany vary strongly. In a review on German programs Küver et al. found 19 different programs for type 2 diabetes, of which only 11 had a formal structure. These 11 programs differed in the methods used and the scope of information and training given [3].

Nevertheless, diabetes self-management education can help to avoid hospitalizations, lower heath care costs and – under certain circumstances – prevent complications like retinopathy, nephropathy, and neuropathy [4]. In spite of these positive effects about 30–50% of the eligible patients do not participate in diabetes education [5]–[6] even though diabetes education is fully compensated by the German statutory health insurance. Similar problems with education attendance are also reported in other countries, e.g. the United States [7] although the reasons for non-attendance might be different between countries.

Several studies from different countries have used a standardized quantitative assessment to identify barriers for diabetes patient education. Barriers identified include time constraints, stress, transportation problems, functional limitations, no interest [8], feeling sufficiently informed [9], and anxious temperament [10]. Until now only few studies have used a qualitative design to identify barriers for diabetes education. One qualitative study identified negative views on diabetes education, group teaching and input from other education participants as barriers [11]. Another study identified experiencing practical problems and feeling insecure as reasons for participation in diabetes education [12].

A previous quantitative study of our research team identified four subgroups of patients refusing to attend diabetes education. These groups were identified by their answers to pre-defined statements concerning non-participation derived from the literature [8]–[12]. In the largest subgroup 50% of these patients denied all response options although all of them felt responsible for their diabetes management and did not feel sufficiently informed. We concluded that these patients might be the best accessible target group for diabetes education, but there seemed to be barriers to attending diabetes education that remained unrevealed in this quantitative design [13].

Therefore the aim of this study was to take a closer look at the patients' attitudes towards diabetes education in order to identify barriers that were not covered by the literature. We assumed that we could gain a better insight into the reasons for not attending self-management education using the patients' own narratives in a qualitative study design. A better knowledge of barriers could help to develop strategies for patient education, e.g. improving recruitment strategies and developing alternative options to group education.

## Methods

We conducted a qualitative study based on semi-structured interviews. The selection of study participants was carried out in cooperation with GP practices. This approach was justified by the fact that the GP usually has a long-standing relationship with his patients and therefore has better chances to recruit the non-attending patients needed for this study than members of the study team. Participation in diabetes education depends on age, level of education, race/ethnicity and place of residence [14]. We expected a variation in the socio-demographic composition of the GP's patient population depending on the location of the practice. For this reason the patient selection was performed in two steps. First, the practices were purposively selected based on their location in different districts of the city. Selection criteria were the variation in socio-demographic variables of the districts [15], i.e. population density, mean income, unemployment rate, percentage of public housing and migrant population. We recruited seven GP practices in Hamburg, Germany. The characteristics of the city districts in which the practices were located are shown in Table 1. Second, we took a convenience sample from the GPs' records in these practices. One of the seven practices was not able to recruit any patients for this study although six patients had been contacted.

**Table 1:** Socio-demographic data of the city districts of the study GP practices.

Practice	District	Population density	Mean income	Unemployment rate	Public housing	Migrant population
A	Volksdorf	1,700/km^2^	50,000€	2.3%	9.2%	13.0%
B*	Rahlstedt	3,300/km^2^	30,000€	5.7%	12.6%	25.6%
C	Eppendorf	8,600/km^2^	45,000€	3.2%	5.1%	17.2%
D	Altona-Altstadt	10,000/km^2^	24,000€	8.5%	20.6%	36.0%
E	Ohlsdorf	2,100/km^2^	30,000€	5.1%	5.9%	18.6%
F	Osdorf	3,500/km^2^	37,000€	7.0%	17.0%	33.3%
G	Eimsbüttel	17,300/km^2^	29,000€	4.3%	2.1%	20.3%
						
	Hamburg total	2,300/km^2^	33,000€	5.9%	11.0%	13.7%

*Practice did not recruit any study participants.

The patients were checked for eligibility criteria and contacted by their GP by telephone or letter if eligible. Eligibility criteria included a diagnosis of type 2 diabetes, enrolment in a disease management program (DMP) for type 2 diabetes and no prior attendance of diabetes education courses. Enrolment in a DMP was required because the German statutory health insurance funds only cover the costs for diabetes education if the patient is enrolled in a DMP. Informed consent was obtained from the study participants prior to the interviews. In total we contacted 30 patients and 18 (60%) of these agreed to participate in our study.

We used the concept of data saturation to determine the number of interviews necessary to obtain valid results. Saturation was assumed when at least four subsequent interviews yielded no additional reasons for non-participation in diabetes education. In a first wave we recruited ten patients, who were interviewed in March and April 2010. Then, in two more waves four patients per wave were recruited and interviewed until saturation was reached. The second wave of patients was interviewed between August and September 2010 and the third in November 2010. We had to exclude four of the 18 patients from data analysis, because they stated during the interviews that they already had participated in diabetes education.

Socio-demographic data of the remaining 14 study participants can be found in Table 2. The patients were between 51 and 81 years old with a mean age of 73 years. Eight study participants were female and six were male. The diabetes duration ranged from nine months to ten years with a mean duration since diagnosis of five years. Six patients had general elementary education or basic vocational qualification, four patients had intermediate qualification or a general maturity certificate, and three patients had lower or higher tertiary education. 13 patients were retired, mostly due to age, and one patient was unemployed.

**Table 2:** Socio-demographic data of study participants and characteristics of interviews.

ID	Gender	Age (years)	Diabetes duration	Education level	Employment status	Int	Time (min)	Location
A1	male	79	3 years	lower tertiary	retired	CK	52	practice
A2	male	78	10 years	intermediate	retired	CK	32	home
C1	female	81	5 years	intermediate	retired	CK	46	home
C2	female	75	4 years	basic vocational	retired	CK	37	home
D1	male	67	4 years	basic vocational	retired	CK	37	café
D2	male	77	*	higher tertiary	retired	CK	32	home
E1	female	62	7 years	maturity certificate	retired	CK	22	home
E2	male	76	10 years	intermediate	retired	CK	24	home
F1	female	52	2 years	basic vocational	unemployed	MP	42	institute
F2	female	73	9 months	basic vocational	retired	MP	41	home
F3	male	78	*	*	retired	MP	40	home
F4	female	68	6 years	basic vocational	retired	MP	39	home
G1	female	74	2 years	general elementary	retired	MP	17	home
G2	female	81	5 years	higher tertiary	retired	MP	63	home

ID: Patient identifier; Int: Interviewer;

* no information provided.

Participants were interviewed face-to-face by CK (a family physician) or MP (a psychologist) at a place of the patient's choice. Most interviews were performed at the patients' home, one interview was conducted at the GP practice, one at our Department of Primary Medical Care and one at an ice cream parlour/café. The interview duration was between 17 and 63 minutes with a mean of 37 minutes.

The interview guideline consisted of a prefatory question concerning the patients' experiences with their diabetes (“please tell me when your diabetes has been diagnosed, the course and how it developed”) followed by several questions concerning the patients' attitudes towards and their knowledge on diabetes education. The audiotaped interviews were transcribed verbatim by trained research assistants. Four researchers were involved in data analysis. MP is a psychologist and psychotherapist in training. IS is a sociologist and involved in health services research about managed care programs and epidemiological research on multimorbidity. HK is a medical doctor with a focus on health services and epidemiological research. NP is a psychologist and expert for qualitative research in the fields of social medicine and health services research.

The researchers performed qualitative content analysis according to Mayring [16]. Data were analysed with Atlas.ti 5.2 software. Categories were determined deductively, i.e. derived in advance from the literature [8]–[12], and inductively, i.e. derived from the interviews. The category system of reasons for non-participation was proposed by MP and IS and discussed by all researchers including HK and NP. The final set of categories was determined by consensus.

The study was carried out in accordance with the Code of Ethics of the Declaration of Helsinki. Patients provided their written informed consent to participate. The study was approved by the Ethics Committee of the Medical Association of Hamburg including the consent procedure (reference number OB-024/07).

## Results

The interview data yielded twelve reasons for non-participation in diabetes education that fell into four themes, namely physician's influence, state of health condition, avoidance and refusal, and knowledge and activity (cf. Table 3).

**Table 3:** Themes and categories of barriers to diabetes education.

Theme	Category
Physician's influence	Physician does not support diabetes education
	Patient is satisfied with diabetes treatment by his physician
	Physician is satisfied with examination results
State of health condition	At the moment the diabetes seems to have no negative consequences
	Comorbidity impedes participation in diabetes education
Avoidance and refusal	Patient avoids listening to narratives of illness
	Patient avoids diabetes education on the behavioural level
	Patient gives diabetes a low priority
	Patient refuses to change his diet
Knowledge and activity	Patient is lacking knowledge about diabetes education
	Patient feels he already knows enough about diabetes
	Patient feels he already does enough about diabetes

### Theme 1: Physician's influence

Our data suggest that the patients' perceptions of statements and behaviour of the attending physician influence the patients' decisions about diabetes education. This especially relates to the physician's support for diabetes education, the quality of the diabetes treatment that the patient receives and the physician's satisfaction with the examination results regarding diabetes.

#### Physician does not support diabetes education

Five patients stated that their physician had not mentioned diabetes education during the consultations, that he had not considered it necessary or that he even had advised against participation. The patients may have refrained from participating in diabetes education, because they relied on this perceived judgment. *“He [my doctor] had told me that I did not need it [a patient education]. I have asked him myself. […] I would need it if I did not adhere to his instructions.” (G2; 81 years old female, 5 years since diagnosis)*

#### Patient is satisfied with diabetes treatment by his physician

Three study participants expressed the feeling that they were being treated very well by their physician. This seems to have led to the impression that the information received during the regular consultations was sufficient for them and that they didn't need additional diabetes education. *“I have never been asked [to participate in a patient education]. […] But I do not have the feeling that I am lacking care. Absolutely not. […] For it [information regarding diabetes] I have my primary care physician. He attends to it.” (E1; 62 years old female, 7 years since diagnosis)*

#### Physician is satisfied with examination results

Seven patients claimed that their attending physician was satisfied with the examination results regarding their diabetes. This positive assessment might have led to the impression that they didn't need to get more active themselves. *“When a blood sample has been taken and I am with her in her office, well then she [my physician] checks her computer: Everything is fine. That is all she says.” (F3; 78 years old male, diabetes duration could not be determined)*

### Theme 2: State of health condition

Patients consider the appraisal of their physician, but they also have their own opinion about their health condition that influences their motivation or ability to attend education measures. In this context the patients especially named a good state of health regarding their diabetes and physical or psychosocial comorbidity as a reason for their non-participation.

#### At the moment the diabetes seems to have no negative consequences

Seven study participants conveyed the impression that their diabetes was still in an early stage or that the blood sugar values were within a normal level. Four of them stressed that they did not experience any negative effects like pain, fatigue or polydipsia. This might have led to the perception that there is no need to attend diabetes education now, and that there still is enough time to attend it in the future. *“As long as my blood sugar is not too high… No, I would not yet attend it [a patient education]. Unless it was rising. Then I suppose I ought to attend it.” (C2; 75 years old female, 4 years since diagnosis)*

#### Comorbidity impedes participation in diabetes education

When it came to their non-participation in diabetes education eight patients referred to physical or psychosocial comorbidity that might have been a barrier. The comorbidities included severe problems with their back or legs, cancer, stroke, chronic ischaemic heart disease, and chronic polyarthritis. Two of the patients reported frequently recurring spells of dizziness so that they were afraid to leave the house. However, in one of these reports the argument might have been pretextual, because the patient later told of a variety of outdoor activities that still were possible. *“[…] I am not going […] to participate in it [the education session], because I also suffer from spells of dizziness. […] And I am not always able to go outside as I would like to do.”* Later in the same interview: *“On days where I notice that I am not well, that I might possibly get a dizzy spell, that's when I do not dare to leave the house. But when I notice: Aha, I feel good, that's when I go outside and walk for an hour. Well, I am also a member of the sports club. And when I am feeling well, that's where I go every Tuesday.” (C2; 75 years old female, 4 years since diagnosis)*

Another two study participants reported major problems with depression that interfered with their motivation for diabetes education. *“Actually, my main problem is more so my mental state. […] What happened during the war, it was so horrible. […] It is only now that it really comes to surface. More and more details. […] There is really nothing else you can do but to get really drunk. […] Well, and of course the situation is such: at my age – at least this is how I look at it – it really does not make a difference to me any longer. Honestly, I do not intend to become one hundred years old. And frankly, I do not really care about any of this.” (D2; 77 years old male, diabetes duration could not be determined)*

### Theme 3: Avoidance and refusal

In most interviews the patients discussed motivational factors of non-participation in diabetes education, in other words if and why they did not want to attend diabetes education. In this context a major theme was avoidance and refusal. In some interviews the patients gave the impression that they did not want to attend an education because they gave their own diabetes a low priority. Other patients seemed to want to attend, but they avoided participation on the behavioural level. There were also a number of patients who seemed to want to avoid certain aspects or side-effects of diabetes education namely the demand of changing their diet and the requirement to listen to narratives of illness of other education participants.

#### Patient avoids listening to narratives of illness

Three study participants stated that one of their main reasons for non-participation in diabetes education was that they did not want to listen to other patients' stories about their illness. On the one hand, we gained the impression that they did not want to get down by other peoples' moaning. On the other hand, they seemed to want to avoid getting frightened by stories of all the bad things that can be caused by diabetes. *“I have also suffered from cervical cancer when I was a young woman. Yes, four surgeries, I stayed at the hospital for almost four months. And afterwards, I continued to receive radiation treatments, a total of one hundred. And you know, […] it was at first every four weeks, then every quarter of a year, then every half a year and every time this chit chat. And the things you had not heard yet and which came to light during this talk. Perhaps many women exaggerate. And I told myself: I do not really want to hear anything about other people's diseases.” (C1; 81 years old female, 5 years since diagnosis)*

#### Patient avoids diabetes education on the behavioural level

Four patients seemed to have realized the benefits of diabetes education and stated that they honestly wanted to participate. Despite this motivation there was something that always kept them from attending. Some of the patients described themselves as too lazy or complacent or they stated that they somehow dodge participating in an education. One study participant told that she herself did not know why she did not participate. Another patient maintained that he tried many times to attend but in the last moment there was always something else that had to be done instead. *“Last time, I almost attended one [an education session] in the previous year. But then we went on vacation. And then I abandoned the idea again. Once we had returned from our vacation there was so much to be done around here. And then there is this and that, and, and, and…” (A2; 78 years old male, 10 years since diagnosis)*

#### Patient gives diabetes a low priority

In contrast, four study participants seemed to give their own diabetes a low priority. One patient maintained that his wife was responsible for his diet, so that he did not want to get an education without her. One patient was unemployed and stated she was too busy doing vocational trainings and did not want any other education at the moment. Another two study participants described they were not in the mood attending an education or that they did not have time to attend, because they had to do other things. *“I really have no time [to attend an education], because I always walk if I don't have to buy something heavy-weight. […] Then I go to the shopping center, […] there is an ice cream parlour where you can sit outdoors. And if the sun is shining, then I take a seat and drink a glass of wine.” (A1; 79 years old male, 3 years since diagnosis)*

#### Patient refuses to change his diet

In our sample two patients reported that they did not want to attend an education because they did not want to change their diet. Maintaining their eating habits seemed to be an essential part of their quality of life. *“My sister-in-law is a diabetic… Well, I am not going to put into my mouth the things she has to eat. I always decide myself what I am eating.” (F3; 78 years old male, diabetes duration could not be determined)*

### Theme 4: Knowledge and activity

The last of the four themes of barriers for diabetes education comprises certain aspects of the patients' knowledge and activity. On the one hand, it seemed to be relevant how much they believed to know about diabetes and to what degree they perceived themselves as self-active and capable regarding this condition. On the other hand, we found that some patients had lacking knowledge about diabetes education that lead to prejudices undermining their motivation to attend.

#### Patient is lacking knowledge about diabetes education

Four patients stated that they did not know exactly what happens during an education but they expressed that they would not attend an education because of a number of negative assumptions they made. These prejudices included that there was too much talk and that it therefore was not really relevant; that the content of a diabetes education was too general and had nothing to do with their specific situation; and that the intellectual level of the education measures was too low for them. *“Often [at a patient education], the general population is overrepresented and I have to listen to things which truly are not any of my concerns. Fact is that they are told not to eat too much and similar things like that. Well, I would not attend it if its standards were not of a somewhat higher level.” (D2; 77 years old male, diabetes duration could not be determined)*

#### Patient feels he already knows enough about diabetes

Four study participants maintained that they already were so well-informed about diabetes from other sources that they did not need a diabetes education. Two of these patients told that their husband had diabetes and that they had gathered their information because of their involvement in his disease management. One patient stated that she received all relevant information from her GP. Another patient referred to his intuition which told him what to do regarding his diabetes. *“‘I have no idea what I am supposed to learn there' expresses it best. […] Because I intuitively stick to my own rules anyway.” (A1; 79 years old male, 3 years since diagnosis)*

#### Patient feels he already does enough about diabetes

Nine patients reported a variety of activities they did regarding their diabetes, including diet, physical exercise, blood sugar self-control, adhering to medication plans and attending examinations by their GP and specialists. They felt that their level of activity was already sufficient and that therefore an education was unnecessary. Additionally, four of these patients stated that they felt confident to manage their disease or that they felt they had their diabetes under control. This might emphasize the impression that diabetes education is not really needed. *“The way I am feeling right now, I really don't need education. I feel good. I have everything under control, don't I?” (C2; 75 years old female, 4 years since diagnosis)*

## Discussion

Our qualitative study aimed to take a closer look at the patients' attitudes towards diabetes education in order to help developing perspectives for patient education in diabetes. We extracted three perspectives from the categories described above. They deal with the questions “How to improve recruitment strategies for diabetes education?”, “Is diabetes education really needed for every patient?” and “Is group education the best way to teach patients with diabetes?”

### How to improve recruitment strategies for diabetes education?

One important factor for participation in diabetes education was the physician's influence. In a previous quantitative study we found a strong association between the recommendation of the attending physician and participation in diabetes education [13]. Other studies have also reported the importance of physician recommendations for increasing participation rates in education measures [17]–[18]. We found in our qualitative approach that this recommendation may be expressed directly by supporting participation in diabetes education, but also in a very subtle form by the level of satisfaction with the diabetes treatment or the examination results expressed during the consultations. Physicians interested in motivating patients for diabetes education should explicitly encourage patients to participate in diabetes education. They should also clarify that patients can profit from education even if diabetes treatment and examination results are promising. However, our results raise the question whether diabetes education is really necessary for all patients with diabetes.

### Is diabetes education really needed for every patient?

Some patients with diabetes have only slightly increased blood sugar values so that they receive a diagnosis, but there are no harmful consequences to be expected at this stage and no treatment is needed. Therefore, participation in an educational program may not be immediately necessary. Another group of patients may already show a sufficient level of knowledge conveyed by family, friends [19], media or their attending physician, and may be active enough to keep their diabetes under control. This patient cluster has also been identified in our quantitative study [13]. As our data are based on self-perception, we have to question the patients' statements about their disease, about their knowledge and activity, because they might not always prove to be true. But if their clinical state and/or their knowledge about diabetes were good, non-participation in diabetes education might be a justified decision. This finding should also be reflected in quality indicators [20]. For this reason studies are needed to quantify how many patients diagnosed with diabetes could profit from diabetes education and how often this is not the case.

### Is group education the best way to teach patients with diabetes?

Diabetes group education might not be the appropriate intervention for all patients in need of education. Some patients refuse to participate in diabetes education because of prejudices and negative feelings regarding group teaching. This phenomenon has been reported before [11]. Others wish to avoid listening to narratives of illness expected in group education, because they do not like listening to other people's moaning or because they fear to hear unpleasant and frightening details about possible complications. There are also practical problems for participating in diabetes education for people with conflicting comorbidity, e.g. functional limitations [8]. In these cases a more individualized approach of education might be a better option than group education, e.g. continuous personal education by a diabetes counselor adapted to the specific situation of the patient. Patients with functional limitations might even need personal education at home. The individualized approach should also relate to the education's content, the intellectual level of the education and the education's methodology. Therefore the physician should try to know about patient's priorities and needs as well as his education level, his knowledge about diabetes and his self-management activities. However, in the German Health Care System this individual education approach needs development and standardization. For example there are training opportunities for nurses to achieve the title “diabetes assistant” or “diabetes consultant”, but reimbursement by the statutory health insurance differs between the 16 federal states of Germany.

### Need for further research

There are some patients who refrained from participating because they did not want to change their diet. This is a paradoxical situation, because patients refusing lifestyle changes might be more willing to comply if they were better informed [21]. Other patients do not take the diabetes seriously or have other priorities in their life. This has already been discussed in our quantitative study [13]. Finally, some patients fail to see the necessity of education, e.g. because they do not experience themselves as ill [21] or they feel they already know and do enough while from the medical point of view there might be still room for improvement. Until now there is no clear concept of how to deal with these barriers. Further research is necessary to better understand these patients and to develop appropriate interventions.

### Strengths and weaknesses

This is the first qualitative study on barriers for diabetes education in Germany. With our qualitative approach we were able to explore barriers for diabetes education that were not yet covered by the literature. The patients were recruited by their GPs, which led to a high response rate of 60%. Our sampling strategy based on sociodemographic variation of the city districts resulted in a sufficient variation in gender, education and diabetes duration of study participants. In contrast, there is little variation in employment status as nearly all patients were retired due to the high age of most patients included in this study. The data analysis was performed by a multidisciplinary team which increased the trustworthiness of the results.

Limitations of this study include the fact that only patient from a large city could be interviewed. For this reason some barriers mainly relevant for patients living in rural areas could have been missed, e.g. transport and distance to the training center. The age-range represented in our study is not unusual, as the percentage of patients with type 2 diabetes increases strongly with age. In the German DEGS1-study 2008/2011 7.2% of the German population aged 18–79 years had a type 2 diabetes. The prevalences according to age groups were: 50–59 years: 5.7%; 60–69 years: 13.8%; 70–79 years: 21.9% [22]. Younger patients might face additional problems not shown in our study like conflicts between work hours and course times. The generalization of the study results to other countries and to patients not enrolled in the DMP might be impaired by the fact that all of our patients had to be enrolled in a DMP. The researchers' own previous work has shown that self-active and motivated patients with a lower risk of diabetic complications seem to be more likely to participate in a DMP [23]. Problems in other countries might also vary from our results because of differences in the health care system (e.g. patients not being referred or not having health insurance coverage).

## Conclusions

Based on the results we draw the following conclusions: Physicians should encourage patients to participate in diabetes education and clarify that they can profit even if treatment and examination results are promising. However, it might be justified that patients do not participate in diabetes education if they only have slightly increased blood sugar values and no risk for harmful consequences or if they already have a sufficient knowledge on diabetes. Additionally, in particular cases, e.g. if patients have other priorities, psychic comorbidity or functional limitations, it might be better to provide continuous personal education adapted to their specific situation instead of group education. The individualized approach should also relate to the education's content, the intellectual level of the education and the education's methodology. In the German Health Care System this individual education approach needs development and standardization.
